# Deciphering the Role of DNA Polymerase Eta on the Incorporation and Bypass of Inosine and Cell Cycle Arrest

**DOI:** 10.3390/ijms262412030

**Published:** 2025-12-14

**Authors:** Jackson C. Lin, Jameson R. Averill, Hunmin Jung

**Affiliations:** Department of Pharmaceutical Science, School of Pharmacy, University of Connecticut, Storrs, CT 06269, USA

**Keywords:** translesion synthesis, DNA polymerase eta, cell cycle arrest, DNA lesion repair and bypass, nucleotide biosynthesis, purine metabolism

## Abstract

Inosine is a key intermediate in many cellular pathways, and our RT-qPCR data showed that DNA polymerase eta (polη) was upregulated upon the repeated treatment of inosine and inosine monophosphate (IMP) in HCT116 cells, which suggests that polη is actively involved in the incorporation and bypass of inosine in cells. To gain novel insight into mutagenic potential of inosine incorporation into DNA and its implication on cell cycle arrest, we conducted structural, biochemical, and cell biological studies of human polη on the incorporation and bypass of inosine. Our nucleotide insertion assay showed that polη incorporated inosine triphosphate (ITP) opposite dC just 18-fold more efficiently than opposite dT, indicating that ITP incorporation by polη is promutagenic. Our three polη crystal structures showed that ITP formed Watson–Crick base pair with dC and that ITP adopted both syn- and anti-conformations across dT, increasing the promutagenicity. Our flow cytometry data showed that only excessive treatment of inosine and IMP caused S- and G2-phase arrest, suggesting that polη’s lesion bypass activity might resolve the cell cycle arrest. Our results give us novel insights into the role of polη in the mutagenic incorporation and bypass of DNA lesions, which might affect cell cycle arrest.

## 1. Introduction

**Figure 1:**
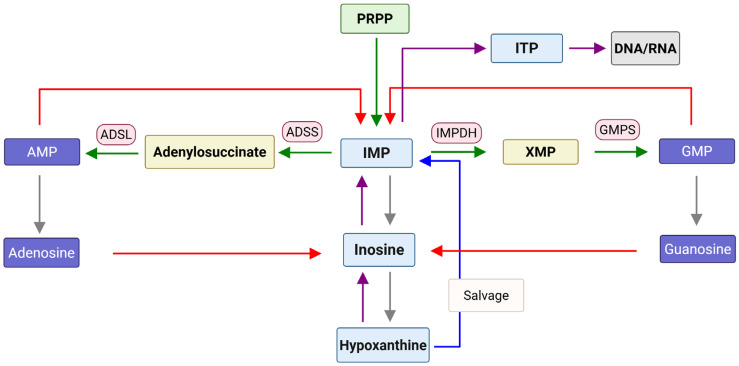
Schematic view of the cellular metabolism of inosine. Green arrows show de novo purine biosynthesis. Purple arrows show the conversion of hypoxanthine into inosine, IMP, and ITP. Red arrows show the conversion of adenosine/AMP and guanosine/GMP into inosine/IMP. Gray arrows show the breakdown of IMP into hypoxanthine.

Inosine, whose base is hypoxanthine (HX), is a key intermediate in many metabolic pathways including purine biosynthesis. Recent studies showed that inosine can be produced by some species in the gut microbiome and is closely related to the immune system and inflammation [1,2]. Inosine monophosphate (IMP) is also a key intermediate in de novo purine nucleotide biosynthesis which generates adenosine monophosphate (AMP) and guanosine monophosphate (GMP) (Figure 1). Inosine can be generated from adenosine through deamination, and this process can be caused by oxidative stress as seen in type 2 diabetes mellitus and potentially other diseases like Alzheimer’s, Parkinson’s, and cardiovascular disease [3]. Increased cellular levels of inosine and its metabolites such as IMP have been shown to be connected to various diseases. The levels of inosine, for example, can be used as a biomarker linked to inflammation, cancer, tumor progression, metastasis, and drug resistance [4]. Inosine levels were also reported to increase in lung squamous cell carcinoma, esophageal squamous cell carcinoma, head and neck squamous cell carcinoma, and bladder cancer patients [5,6]. Adenosine and inosine are known to permeate the blood–brain barrier, and a change in plasma/serum levels may reflect a change in central nervous system (CNS) and systemic inosine administration and the increased level can affect the CNS [7]. Increased levels of inosine were observed in the cerebrospinal fluid of acute and chronic pain patients [8] and in the serum of fibromyalgia patients [9]. Inflammation is also reported to be related to the increased levels of inosine in mouse model [10], and increasing extracellular inosine, either directly by inosine intake or pharmacological inhibition of cellular inosine transporters (indirect), increases whole-body energy expenditure and can counteract obesity [11]. The increased levels of inosine were reported to lead to an increase in dIMP/IMP and dITP/ITP levels, which can result in the misincorporation of inosine into DNA and RNA [12,13] (Figure 1).

The human genome is under constant attacks by a wide variety of exogenous and endogenous damaging agents. One of the most abundant DNA damages in eukaryotes, including humans, is ribonucleotide (rNP), and the incorporation of rNP is sometimes even performed by replicative DNA polymerases [14,15]. Though the presence of rNPs in DNA was identified in the 1970s in mammalian mitochondrial genomes [16], it was only recently that information on how prevalent they are in genomes, how they are removed, and what immediate consequences they have in mammals were discovered [17,18,19]. Many DNA polymerases are known to select deoxyribonucleotides over rNPs using a steric hindrance in their active sites between 2′-OH in the ribose ring of the incoming rNPs and the specific residue(s) playing a role as a gate [20], and Phe18 was reported to be the gate residue in DNA polymerase eta (polη) [21].

To study the incorporation of rNP, especially inosine, into DNA and their mutagenic potential in cells, we chose one of the Y-family DNA polymerases, human DNA polymerase η (polη), which plays a crucial role in translesion DNA synthesis (TLS). Polη was reported to efficiently bypass a wide range of DNA lesions including xanthine and hypoxanthine [22], 8-oxoguanine (oxoG) [23], 8-oxoadenine (oxoA) [24,25], N7-alkylguanines [26,27,28,29], and the contributing factors that affect the mutagenic lesion bypass by polη have been well compiled in our recent review [30]. In addition, we recently reported that polη is involved in not only the bypass but also the direct incorporation of DNA lesions such as 5-fluorouracil (5-FU) [31]. However, the structural and biochemical basis for the mutagenic potential of the incorporation of many other lesions, including inosine ribonucleotide, into DNA remains poorly understood.

Polη has been known for its unique ability to accommodate ribonucleotide across DNA and RNA templates. Several thousands to millions of ribonucleotide monophosphates (rNMPs) are reported to be sporadically present among the DNA bases of eukaryotic genomes [14]. The 2′-OH group in the ribose ring makes the sugar phosphate backbone of RNA 100,000-fold more prone to spontaneous hydrolysis than DNA, and ribonucleotides (especially rNMPs) induce B-form to A-form transition in the double helix, potentially affecting protein–DNA interactions and the catalytic activity of various DNA-interacting enzymes including DNA polymerases, topoisomerases, and ligases, and leading to further DNA damage [32]. rNMPs in the genome constitute one of the most abundant DNA lesions in eukaryotic cells [33,34], and sequencing studies in yeast displayed that cytosine and guanosine were the most abundant rNMPs in the genome [35,36]. Incorporation of rNMPs into the genome of proliferating cells is predominantly mediated by the replicative Pols δ and ε [14], but there have been reports on the incorporation of ribonucleotides by other specialized DNA polymerases such as polβ [37], polμ [38], polλ [39], polι [40], and polη [21,41]. rNMP incorporation during DNA repair or translesion synthesis (TLS) is a likely event in vivo, given that the intracellular ribonucleoside triphosphates (rNTPs) concentrations are 100- to 1000-fold higher than those of deoxyribonucleoside triphosphates (dNTPs), and rNMPs incorporation opposite a DNA lesion may delay subsequent removal of either the rNMPs or the damaged base in the context of TLS [21,42,43]. Polη was reported to incorporate ribonucleotides across undamaged DNA and lesion-containing DNAs such as CPD or 8-oxoG [21,41,44]. Though the incorporation of ribonucleotides can be performed by polη, the efficiency was quite low compared to the incorporation of deoxyribonucleotides. For example, polη was reported to bypass dCTP across the undamaged dG about 780 times more efficiently in terms of *k*_cat_/*K*_m_ compared to CTP bypass [21].

Herein, we report reverse transcription quantitative PCR (RT-qPCR) data for mRNA gene expression upon the treatment of inosine and inosine monophosphate (IMP) in the HCT116 cell line, a human colorectal cancer cell line. We also report flow cytometry analysis data for the changes in cell cycle percentages upon inosine and IMP treatments in HCT116 cells. Human polη kinetic data via nucleotide insertion assay for the incorporation of ribonucleotide of inosine (ITP) opposite templating dC and dT along with the crystal structures of polη in complex with the incoming ITP across the templating dC and dT. These human DNA polymerases-based studies will provide new insights into the mutagenic potential of inosine triphosphates and their implication in replication and translesion synthesis.

## 2. Results

### 2.1. Polη and Polδ Were Upregulated upon the Treatment of Inosine and IMP in HCT116 Cell Line

**Figure 2:**
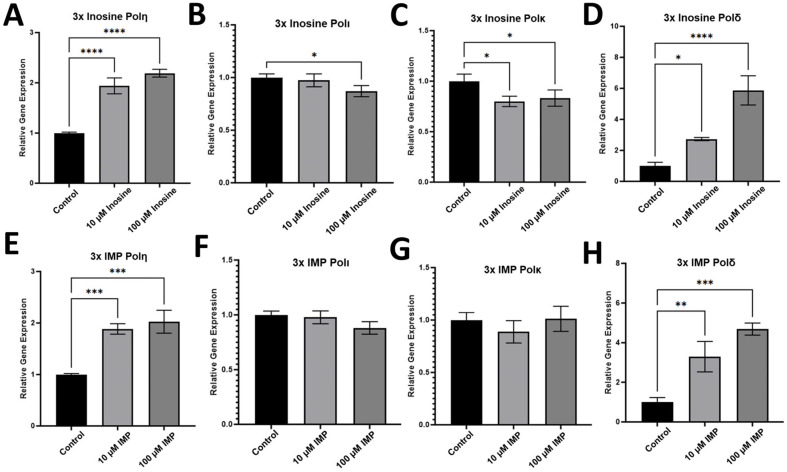
RT-qPCR data for the gene expression in HCT116 cell line. Gene expression upon the 3x treatments of inosine (**A**–**D**) and IMP (**E**–**H**) with 72 h intervals between the treatment. Among the translesion synthesis (TLS) polymerases, (**A**,**E**) polη, (**B**,**F**) polι, (**C**,**G**) polκ, only polη was upregulated upon the treatment of inosine and IMP. The replicative DNA polymerase, (**D**,**H**) polδ, was also upregulated upon the treatment of inosine and IMP. Statistical significance is indicated as follows, * *p* ≤ 0.05, ** *p* ≤ 0.01, *** *p* ≤ 0.001, **** *p* ≤ 0.0001, and *p*-values from one-way ANOVA are less than 0.001 for all the genes except for polκ whose *p*-value is 0.002 (n = 3).

To evaluate whether the administration of inosine and its metabolite, IMP, and the misincorporation of inosine into DNA affect the gene expression in the HCT116 cell line, we performed RT-qPCR with the mRNA samples extracted from HCT116 cells treated with 10 and 100 μM inosine (Figure 2A–D) or IMP (Figure 2E–H). According to the RT-qPCR experiments with the mRNA extracted from HCT116 cells, polη, which is one of the main DNA polymerases employed in TLS, was upregulated by the 3x treatment of both inosine (Figure 2A) and IMP (Figure 2E) at both 10 and 100 μM. Interestingly, there was almost no difference in the level of upregulation of polη in the two concentrations of inosine or IMP despite the 10-fold difference (10 vs. 100 μM). Similarly, one of the replicative DNA polymerases, polδ, was also upregulated by the treatment of both inosine (Figure 2D) and IMP (Figure 2H) at 10 and 100 μM. However, unlike polη, the upregulation level was much higher in 100 μM treatment than 10 μM in both inosine and IMP treatments, suggesting that HCT116 cells utilize polη and polδ differently in response to the elevated level of inosine and its metabolites.

### 2.2. Other TLS Polymerases Were Not Significantly Upregulated upon the Treatment of Inosine or IMP

While polη was upregulated upon the triple treatments of inosine and IMP, other TLS polymerases, including DNA polymerase iota (polι) and kappa (polκ), were not significantly upregulated upon the same treatment of inosine (Figure 2B,C) and IMP (Figure 2F,G). This is an intriguing finding in that this might indicate polη is the only TLS polymerase that can incorporate and bypass inosine, while the other major TLS polymerases including polι and polκ were not engaged in the incorporation and/or bypass of inosine in HCT116 cells. Further research analyzing inosine bypass or ITP incorporation by polι and polκ in other cancer cells will confirm if this is the case for cancer in general. It is worth noting that a replicative DNA polymerase, polδ, is also involved in the incorporation of inosine, and it remains to be seen if there is any mechanism/circumstance to differentiate which DNA polymerase between polδ and polη for the incorporation of inosine into DNA. In HCT116 cells, polη was the only TLS polymerase that was upregulated in response to the treatments of inosine or IMP, and there were almost no responses from polι and polκ upon the same treatments.

### 2.3. Inosine and IMP 5x Treatments Induced S- and G2-Phase Arrest in Replication in HCT116 Cells

**Figure 3:**
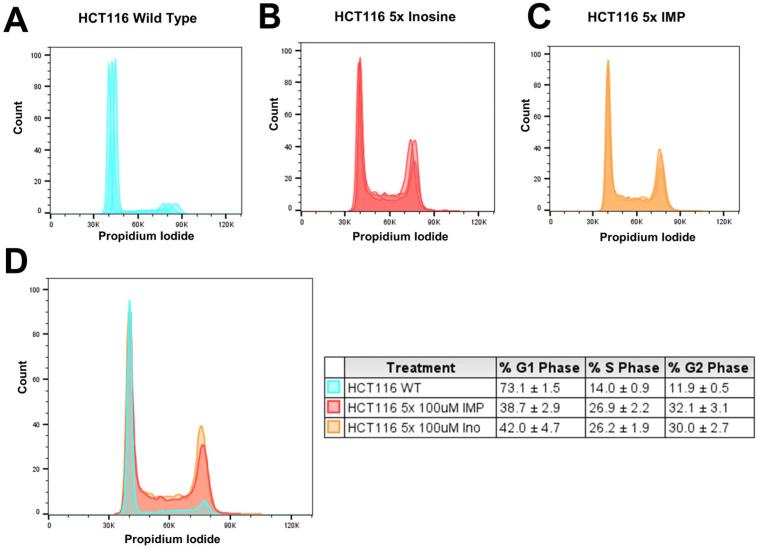
Flow cytometry analysis of cell cycle population upon the treatment of inosine and IMP in HCT116 cells. Compared to the untreated cells (**A**), the inosine-treated (**B**) and IMP-treated cells (**C**) showed an increased S- and G2-phase population. When the three graphs were superimposed (**D**), these changes are clearly shown with the exact percentages of the cell cycle presented in the box. All the experiments were triplicated (n = 3), and the averages and standard deviations are shown in the box.

To examine the effect of the treatments of inosine and IMP on the cell cycle during the replication, we tested HCT116 cells which were treated 1, 3, and 5 times with inosine or IMP via flow cytometry. There was no cell cycle arrest effect from 1x or 3x treatments of inosine or IMP, and only 5x treatment showed an increased population of S- and G2-phase (Figure 3). Compared to no treatment (Figure 3A), the 5-time treatment of inosine (Figure 3B) increased the S-phase (From 14.0% to 26.1%) and G2-phase (From 11.9% to 30.0%) percentages. Similarly, the 5-time treatment of IMP (Figure 3C) also increased the S-phase (From 14.0% to 26.9%) and G2-phase (From 11.9% to 32.3%) percentages. In both inosine and IMP treatments, the S-phase was increased about 12% and the G2-phase was increased about 20% compared to the untreated wild type (Figure 3D). This result indicates that the increased amount of inosine and IMP, which are usually metabolized or repaired, in DNA and cells could induce S- and/or G2-phase arrest in replication, which can lead to cell death.

### 2.4. Incorporation of ITP Across dC by Polη Is More Efficient than GTP or ATP Incorporation

**Figure 4:**
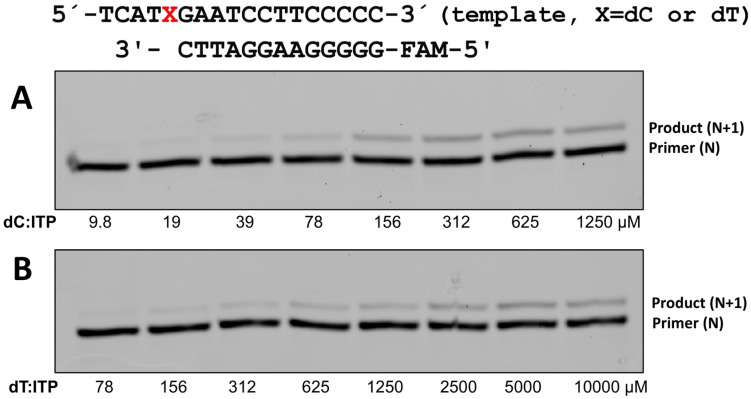
The incorporation of ribonucleotide of inosine triphosphate (ITP) by polη. ITP incorporation opposite templating X in red, which is representative of dC (**A**) or dT (**B**). Primer-template dsDNA was mixed with polη, and the reactions were initiated by the addition of 8 different concentrations of ITP. 5′-FAM-labeled primer was used for the single nucleotide incorporation study, and the reaction was conducted for 4 (dC) or 6 (dT) minutes at 37 °C and the quenched samples were separated on 18% denaturing polyacrylamide gels.

To evaluate whether the incorporation of ITP by polη is efficient and promutagenic, we first determined the kinetic parameters for polη incorporating ITP opposite a templating dC and dT along with the control incorporation of purine ribonucleotides (ATP and GTP) across dC or dT (Table 1 and Figure 4). Inosine is formed from adenosine via deamination in cells, and the ATP incorporation across dC can be a control for ITP incorporation across dC. The catalytic efficiency (*k*_cat_/*K*_m_) of ITP incorporation opposite dC by polη is 0.02, and the catalytic efficiency of ITP incorporation is about 360 times less efficient than that of dITP insertion opposite dC (0.02 for ITP vs. 7.17 for dITP). This might seem quite low, but it is much higher than the ribonucleotide incorporation of guanine or adenine. For example, the *k*_cat_/*K*_m_ value for GTP incorporation across dC is 0.0073, while the *k*_cat_/*K*_m_ value for dGTP incorporation across dC is 18.5, which is about a 2540-fold difference. This result highlights that polη-mediated inosine ribonucleotide is much more efficient than the incorporations of the canonical ribonucleotides, especially compared to the incorporations of deoxyribonucleotides.

### 2.5. Polη Was Able to Incorporate ITP Across dT That Can Cause High Mutagenicity

To evaluate whether the incorporation of ITP by polη is promutagenic, we also determined the kinetic parameters for polη incorporating ITP opposite a templating dT (Table 1 and Figure 4). For the incorporation of ribonucleotide, the incorrect insertions of canonical ribonucleotides (e.g., ATP insertion across dC) by polη were shown to be not efficient enough to be detected on gels (Table 1). On the other hand, polη was able to incorporate ITP across dT with less efficiency compared to the incorporation across dC (*k*_cat_/*K*_m_ being 0.0011 for across dT vs. 0.02 for across dC) and this ratio of 18:1 between correct and incorrect insertion is slightly higher than the same ratio in dITP incorporation (14:1). Overall, the incorporation of ITP by polη was shown to be promutagenic compared to the incorporation of canonical ribonucleotides.

### 2.6. ITP Formed a Non-Optimal Watson–Crick Base Pair with the Templating dC

Our kinetic studies showed that polη incorporated ITP opposite the templating dC more efficiently than other ribonucleotides’ (ATP or GTP) incorporation (Table 1). To acquire insights on the structural features of ITP incorporation into DNA by polη, we elucidated a crystal structure of polη complexed with a recessed dsDNA containing dC with the incoming ITP. The polη-dC:ITP ternary complex was crystallized in P6_1_ space group with the cell dimension of a = 99.27 Å, b = 99.27 Å, c = 81.76 Å, α = 90.00°, β = 90.00°, and γ = 120.00°. The polη-dC:ITP ternary structure was refined to a resolution of 1.64 Å with R_work_ = 20.9% and R_free_ = 24.1% (Table 2).

**Figure 5:**
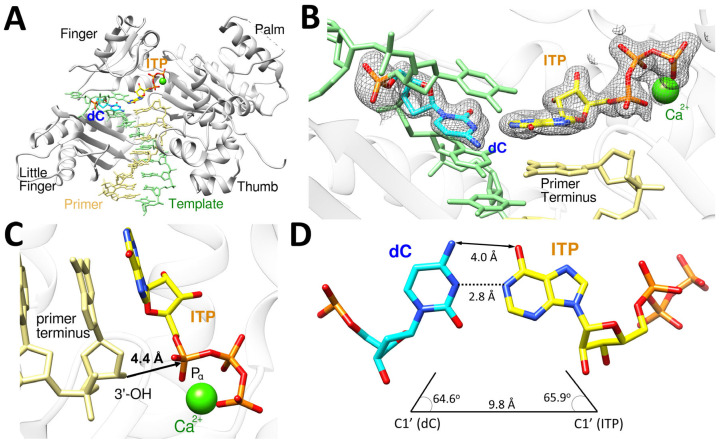
The ternary structure of polη complexed with a recessed dsDNA with templating dC and the incoming ITP. (**A**) Overall structure of polη complexed with the templating dC and the incoming ITP in the presence of Ca^2+^. All four subdomains, finger, little finger, thumb and palm, are conserved. (**B**) The close-up view of the active site of the polη-dC:ITP ternary structure. The 2*F*_o_−*F*_c_ electron density around dC and ITP contoured at 1σ is shown and is well ordered in the active site of polη. (**C**) The incoming nucleotide and metal binding site of polη-dC:ITP displays that 3′-OH of the primer is 4.4 Å away from P_α_ of ITP with one Ca^2+^ ion well positioned in the active site as well. (**D**) ITP and dC formed Watson–Crick base pair, and the geometry of the base pair is close to the optimal Watson–Crick base pair.

The polη-dC:ITP ternary complex structure provides the structural basis for correct incorporation of ITP opposite dC by polη (Figure 5), and this structure displays the conserved secondary structures of polη and the four characteristic domains (thumb, palm, finger, and little finger) of Y-family DNA polymerases (Figure 5A). The dC:ITP base pair is well ordered and accommodated in the catalytic active site of polη, as indicated by the strong and well-defined electron density (2*F*_o_−*F*_c_ = 1σ) around dC and the incoming ITP with one calcium ion present (Figure 5B). The primer terminus 3′-OH is coordinated to the A-site calcium ion and is about 4.4 Å away from the Pα of ITP (Figure 5C) being positioned for in-line nucleophilic attack on the Pα of the ITP. Though the base pair between bases of dC and ITP is slightly off of the canonical Watson–Crick base pair due to the presence of 2′-OH, the templating dC displayed Watson–Crick-like geometry in base pairing with the incoming ITP, which is similar to the canonical Watson–Crick base pair, with the distances of 4.0 Å (N4 of dC and O6 of ITP) and 2.8 Å (N3 of dC and N1 of ITP) between them (Figure 5D). Unlike canonical Watson–Crick base pair, the slightly longer distance (4.0 Å) between N4 of dC and O6 of ITP, which makes it a van der Waals interaction instead of a hydrogen bonding interaction, was observed because hypoxanthine ring of ITP is not in the same plane with dC due to the presence of 2′-OH (Figure 5B). The geometry of dC:ITP base pair displayed the λ angles of 64.6° (dC) and 65.9° (ITP) and the C1′-C1′ distance of 9.8 Å (Figure 5D), which is close to that of the canonical undamaged base pairs.

### 2.7. ITP Adopted Either Anti- or Syn-Conformation to Form a Base Pair with the Templating dT in the Active Site of Polη

Our kinetic studies showed that polη less efficiently incorporated ITP opposite the templating dT compared to the templating dC, yet ITP incorporation across dT by polη, which would cause both ribonucleotide lesion and A:T to G:C mutation, was the only incorrect ribonucleotide incorporation in our experiment (Table 1). To gain structural insights on how polη incorporates ITP opposite dT, while GTP across dT or ATP across dC were not observed, we elucidated crystal structures of polη complexed with recessed dsDNA-containing dT with the incoming ITP. Two distinct structures were elucidated, and it turned out that one polη-dT:ITP ternary structure contained anti-conformation of ITP and the other structure contained syn-conformation of ITP. This heterogeneity of base pair between ITP and the templating dT might be the reason why ITP incorporation across dT was able to happen, while GTP incorporation across dT or ATP incorporation across dC was not.

**Figure 6:**
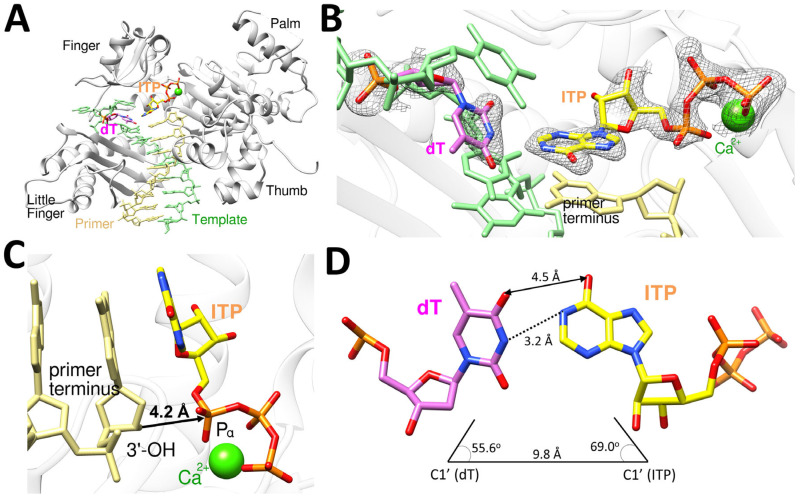
Ternary complex structure of polη complexed with a recessed dsDNA with templating dT and the incoming ITP (anti). (**A**) Overall structure of polη complexed with templating dT and the incoming ITP (anti) in the presence of Ca^2+^ with all four subdomains, finger, little finger, thumb and palm, are all shown. (**B**) Close-up view of the active site of the polη-dT:ITP (anti) ternary structure. The 2*F*_o_−*F*_c_ electron density map around dT and ITP contoured at 1σ is shown in the active site of polη. (**C**) The incoming ITP and metal binding site of polη-dT:ITP displays that 3′-OH of the primer is 4.2 Å away from P_α_ of ITP with just one Ca^2+^ ion in the active site. The orientation of 3′-OH is in the non-optimal direction and distance for the nucleophilic attack on P_α_ of the incoming ITP. (**D**) The base pair between dT and ITP is a Watson–Crick-like base pair, with the hydrogen bonding interaction between N3 of dT and N1 of ITP, along with van der Waals interaction between O4 of dT and O6 of ITP.

The polη-dT:ITP (anti) ternary complex was crystallized in P6_1_ space group with the cell dimension of a = 98.90 Å, b = 98.90 Å, c = 81.22 Å, α = 90.00°, β = 90.00°, and γ = 120.00°. The polη-dT:ITP (anti) ternary structure was refined to a resolution of 1.74 Å with R_work_ = 21.1% and R_free_ = 24.5% (Table 2). The polη-dT:ITP (anti) ternary complex structure provides a structural basis for the incorrect incorporation of ITP opposite dT by polη (Figure 6). This structure, similar to polη-dC:dITP, displays the conserved secondary structures and the four characteristic domains (thumb, palm, finger, and little finger) of Y-family DNA polymerases (Figure 6A). The dT:ITP (anti) base pair is well ordered and accommodated in the catalytic active site of polη, as indicated by the well-defined electron density (2*F*_o_*−F*_c_ = 1σ) around dT and the incoming ITP (Figure 6B). The structure showed just one catalytic metal in the active site with one calcium missing at A-site, which is close to 3′-OH of the primer terminus, and the primer terminus 3′-OH has no coordination with a metal due to the missing A-site calcium ion and displayed about 4.2 Å distance from the Pα of ITP (Figure 6C), being less optimally positioned for in-line nucleophilic attack on the Pα of the ITP. The templating dT formed a Watson–Crick-like base pair with ITP (anti) via 4-enol-2-keto tautomer of thymine with the inter-base hydrogen bonding distance of 3.2 Å (N3 of dT and N1 of ITP), along with van der Waals interaction between O4 of dT and O6 of ITP with the distance of 4.5 Å (Figure 6D). The geometry of dT:ITP (anti) base pair displayed the λ angles of 55.6° (dT) and 69.0° (ITP) and the C1′-C1′ distance of 9.8 Å (Figure 6D), which is not significantly different from that of correct undamaged base pairs.

### 2.8. Syn-ITP Formed Wobble Base Pair with the Templating dT in Polη

Our structural studies on the polη-dT:ITP ternary complex showed that polη incorporated ITP across dT in two distinct fashions via anti- and syn-conformations of ITP. To gain insights on the structural features of ITP incorporation opposite dT by polη, we also solved a crystal structure of polη complexed with recessed dsDNA-containing dT with the incoming ITP, which was found to be a syn-conformation in this structure. The polη-dT:ITP ternary complex was crystallized in P6_1_ space group with the cell dimension of a = 99.30 Å, b = 99.30 Å, c = 81.61 Å, α = 90.00°, β = 90.00°, and γ = 120.00°, and this polη-dT:ITP (syn) ternary crystal structure was refined to a resolution of 2.11 Å with R_work_ = 20.5% and R_free_ = 25.7% (Table 2).

**Figure 7:**
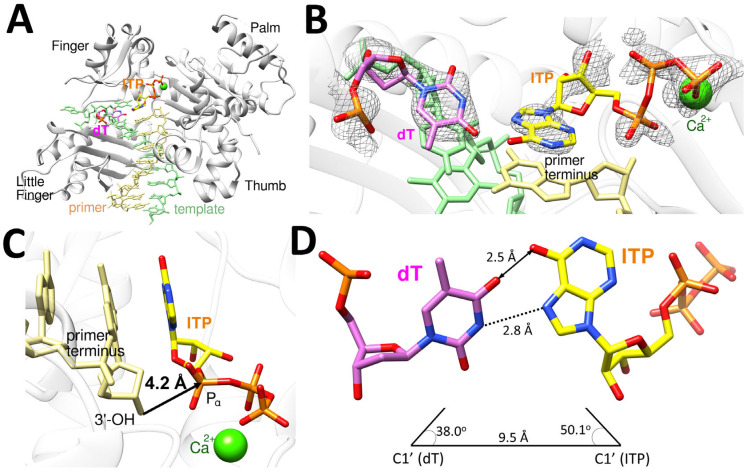
Ternary complex structure of polη complexed with a recessed dsDNA with templating dT and the incoming ITP (syn). (**A**) Overall structure of polη complexed with templating dT and the incoming ITP in the presence of Ca^2+^ with all four subdomains, finger, little finger, thumb and palm, are all shown. (**B**) Close-up view of the active site of the polη-dT:ITP (syn) ternary structure. The 2*F*_o_−*F*_c_ electron density around dT and ITP contoured at 1σ is shown in the active site of polη. (**C**) The incoming nucleotide and metal binding site of polη-dT:ITP (syn) displays that 3′-OH of the primer is 4.2 Å away from P_α_ of ITP with just one Ca^2+^ ion in the active site. The orientation of 3′-OH is in the non-optimal direction and distance for the nucleophilic attack on P_α_ of the incoming ITP. (**D**) The base pair between dT and ITP (syn) is a wobble base pair, with the hydrogen bonding interaction between N3 of dT and N7 of ITP, along with the van der Waals interaction between O4 of dT and O6 of ITP.

The polη-dT:ITP (syn) ternary complex structure provides the structural basis for how the incorrect incorporation of ITP opposite dT can be the only incorrect ribonucleotide incorporation by polη (Table 1, Figure 7). This structure, similar to polη-dT:ITP (anti), displayed the conserved secondary structures and the four characteristic domains (thumb, palm, finger, and little finger) of Y-family DNA polymerases (Figure 7A). The dT:ITP (syn) base pair is less ordered compared to dC:ITP or dT:ITP (anti) and is accommodated in the catalytic active site of polη, as indicated by the electron density (2*F*_o_*−F*_c_ = 1σ) around dT and the incoming ITP (syn) (Figure 7B). The polη-dT:ITP (syn) ternary complex structure showed one catalytic metal in the active site with one calcium missing at A-site, and the primer terminus 3′-OH has no coordination with a metal due to the missing A-site calcium ion and displayed about 3.9 Å distance from the Pα of ITP (Figure 7C), being less than optimally positioned for in-line nucleophilic attack on the Pα of the ITP. All three structures we present here showed just one calcium ion in the active site, and it might be a common phenomenon for a ribonucleotide insertion by polη. The templating dT formed a wobble base pair with ITP with an inter-base hydrogen bonding with the distance of 2.8 Å between N3 of dT and N7 of ITP, along with van der Waals interaction between O4 of dT and O6 of ITP with the distance of 2.5 Å (Figure 7D). The geometry of dT:ITP base pair displayed the λ angles of 38.0^o^ (dT) and 50.1^o^ (ITP) and the C1′-C1′ distance of 9.5 Å (Figure 7D), which is somewhat different than the canonical base pairs.

### 2.9. Expression of the Genes in Nucleotide Biosynthesis and DNA Polymerase β upon the Treatment of Inosine and IMP

**Figure 8:**
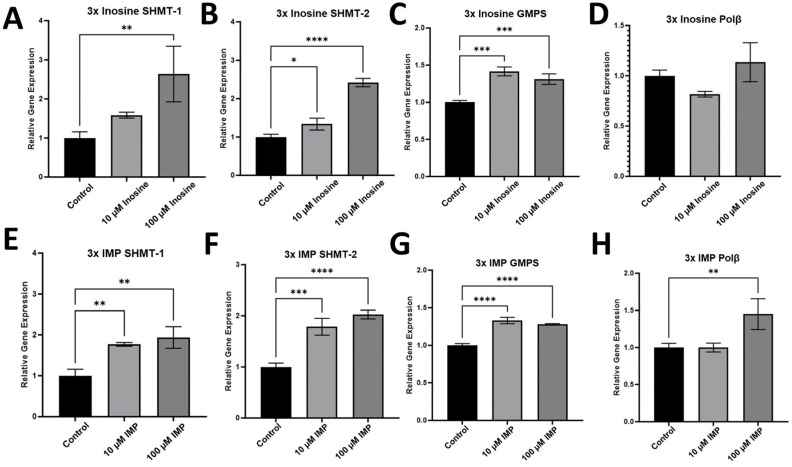
RT-qPCR data for the gene expression of non-TLS genes in HCT116 cell line. Gene expression after the 3-time treatments of inosine (**A**–**D**) and IMP (**E**–**H**) with 72 h intervals between the treatment. Among the genes in nucleotide biosynthesis, SHMT-1 (**A**,**E**), SHMT-2 (**B**,**F**), and GMPS (**C**,**G**) were checked, and SHMT-1 and 2 were upregulated upon the treatment of inosine and IMP. Another DNA polymerase, polβ, was not significantly upregulated upon the treatment of inosine (**D**) and slightly upregulated upon the treatment of 100 µM IMP (**H**). Statistical significance is indicated as follows, * *p* ≤ 0.05, ** *p* ≤ 0.01, *** *p* ≤ 0.001, **** *p* ≤ 0.0001, and *p*-values from one-way ANOVA are less than 0.001 for all the genes (n = 3).

To evaluate whether the administration of inosine and IMP affects the expression of the genes in nucleotide biosynthesis and base excision repair (BER) in the HCT116 cell line, we performed RT-qPCR with the mRNA samples extracted from HCT116 cells treated with 10 and 100 μM inosine (Figure 8A–D) or IMP (Figure 8E–H). Our RT-qPCR data showed that there are some other genes, in addition to polη, that were upregulated upon the treatment of inosine or IMP in the HCT116 cell line. Upon the 3x treatment of inosine and IMP, serine hydroxymethyltransferase (SHMT) 1 (Figure 8A,E) and 2 (Figure 8B,F), which are crucial enzymes in one-carbon metabolism and pyrimidine biosynthesis in cytosol (SHMT-1) and in mitochondria (SHMT-2), were upregulated in both 10 and 100 μM concentrations of inosine with higher expressions in 100 μM treatments. SHMT was the only enzyme in the thymidylate cycle that was upregulated upon the treatment of inosine or IMP. Guanosine monophosphate synthase (GMPS) is one of the enzymes in purine biosynthesis (Figure 1), and GMPS was slightly upregulated upon the treatment of inosine or IMP (Figure 8C,G). It is intriguing to see that the increased level of inosine or IMP increased the gene expression of SHMT without affecting the expression of GMPS, and this might indicate that the externally added inosine or IMP do not engage directly in the purine biosynthesis, but rather become metabolized first in cells. Another notable enzyme is polβ, which is encoded by *POLB* gene and is one of the key enzymes in BER, and the expression levels of polβ in melanoma, colon, and breast cancers were reported to be significantly higher compared with the adjacent normal tissues [46,47], and polβ can extend DNA at a concentration as low as 5 nM [24]. The expression of polβ was slightly upregulated upon the treatment of IMP (Figure 8H) but not upon the treatment of inosine (Figure 8D). In addition, other genes in BER, such as uracil DNA glycosylase-1 (UDG-1) or Methyl-CpG binding domain protein 4 (MBD4), were not significantly up- or downregulated upon the treatment of inosine or IMP.

## 3. Discussion

### 3.1. Polη Is One of the First Responders for the Increased Level of Inosine/IMP Which Efficiently Incorporates Inosine into DNA

Our RT-qPCR data showed that polη and polδ are the only two DNA polymerases that were upregulated upon the 3x treatments of inosine or IMP, and other enzymes in TLS, including polι or polκ, were not upregulated (Figure 2). This means that polη along with the replicative DNA polymerase, polδ, can incorporate inosine into DNA when the cellular level of inosine is elevated. Since inosine is known to act like guanine in DNA, inosine incorporation can cause A:T to G:C mutation. We recently showed that polη is involved in the incorporation of inosine into DNA via deoxyinosine triphosphate (dITP) [45], and our data presented here showed that polη can also incorporate ribonucleotide of inosine into DNA as well. The catalytic efficiency in terms of *k*_cat_/*K*_m_ of polη for the incorporation of ITP is about 360 times less efficient than that of dITP insertion opposite dC (0.02 for ITP vs. 7.17 for dITP, Table 1), and this ratio is remarkably low compared to the same ratio for guanine or adenine incorporation. The catalytic efficiency (*k*_cat_/*K*_m_) of polη for ATP incorporation across dT is 0.0048, while the *k*_cat_/*K*_m_ of polη for dATP incorporation across dT is 19.9 (Table 1), which is about a 4150-fold difference. The same ratio for the incorporation of GTP and dGTP by polη was a 2540-fold difference (Table 1). These results suggest that polη is employed first upon the increase in cellular inosine level to incorporate inosine into DNA along with the bypass of the inosine lesion, and there should be more investigation performed on how polη differentiates its role of bypass and incorporation of inosine in cells.

The three crystal structures of polη complexed with ITP across dC and dT (anti and syn) we presented here also give valuable insights into the inosine incorporation across the canonical pyrimidine bases. The correct insertion of ITP across dC formed the similar Watson–Crick base pair (Figure 5) shown in dC:dITP, except for the bigger propeller plane between dC and ITP due to the presence of 2′-OH on the ribose ring of inosine. This structural similarity in the base pair of ITP and dITP across dC can lead to the higher reaction efficiency, compared to GTP or ATP incorporation, of the correct incorporation of ITP across dC by polη (Table 1). Ribonucleotide incorporation is one of the most frequent DNA lesions and is performed by some DNA polymerases including polη during replication [48], and the incorporated ribonucleotides are often removed by DNA repair pathways including ribonucleotide excision repair (RER), which is initiated by RNase H2 [33].

### 3.2. Incorporation of ITP by Polη Is Promutagenic via Anti-Syn Conformational Heterogeneity of ITP

Our kinetic data revealed that polη was able to incorporate inosine ribonucleotide across dT, which is an incorrect insertion plus ribonucleotide insertion, while ATP incorporation across dC or GTP incorporation across dT were not observed (Table 2). The ratio of the catalytic efficiency in terms of *k*_cat_/*K*_m_ between the incorporation of ITP across dC and across dT is around 18:1 (*k*_cat_/*K*_m_ being 0.02 for dC:ITP and 0.0011 for dT:ITP), and this ratio is similar with the ratio of 14:1 between the incorporation of dITP across dC and dT (*k*_cat_/*K*_m_ being 7.17 for dC:dITP and 0.52 for dT:dITP). The crystal structure of polη complexed with the templating dT and the incoming ITP (syn) gives us an insight into this promutagenic incorporation of ITP by polη. The polη-dT:ITP ternary complex structure showed that ITP can adopt both anti- (Figure 6B) and syn- (Figure 7B) conformation in the active site of polη, and this heterogeneity in the base pairing between ITP and dT enables polη to efficiently incorporate ITP across dT, which was not observed in canonical purine ribonucleotides including ATP or GTP. Syn–anti conformational change was also shown to be one of the contributing factors for the promutagenic bypass of some lesions including N7-alkylguanine by polη [28,30], and our study here provides additional insight into the effect of the conformational heterogeneity of the nucleotides on the promutagenic potential of DNA polymerases. When inosine is present in DNA, the bypass of the inosine (HX) lesion by polη was shown to be almost exclusively dCTP incorporation (the ratio of *k*_cat_/*K*_m_ for the incorporation across HX by polη between dCTP and dTTP was 70:1, Table 1). On the other hand, the incorporation of ITP and dITP by polη was shown to be more promutagenic, with the same ratio of *k*_cat_/*K*_m_ being 18:1 and 14:1, respectively. This might indicate that inosine is more mutagenic in the incorporation stage than the bypass when polη and TLS are involved, and it remains to be seen if there is any inherent mechanism for polη to differentiate its involvements in the incorporation or the bypass of inosine. While polη kinetics support the promutagenic effect of inosine/IMP, the mutational burden via cell-based experiments still needs to be determined. It should also be investigated what the implication of this incorrect ribonucleotide incorporation of ITP across dT is in cancer cells, where cells grow faster than normal cells and the need for nucleotide is greater for the completion of DNA replication, and many non-replicative DNA polymerase, including polη and polβ, engage in the DNA incorporation and bypass of DNA lesions including inosine to ensure the completion of DNA replication, which can cause many incorrect incorporations.

Our data presented here give novel insight into the roles of polη and TLS in generating DNA lesions including ribonucleotides and bypassing those lesions, and we recently showed that polη can incorporate 5-flouro-uracil (5-FU) into DNA as well [31]. It is enigmatic to see that a DNA polymerase is used to incorporate a lesion into DNA and to bypass the same lesion, and several research groups including our lab are working on when and how the ribonucleotide incorporation into DNA happens during replication. The incorporation of inosine by polη we showed here can be a good starting point for in-depth investigations on the ribonucleotide lesions that are generated from DNA-incorporating drugs.

### 3.3. Polη Is a Potential Modulator for the S- and G2-Phase Cell Cycle Arrest During Replication

**Figure 9:**
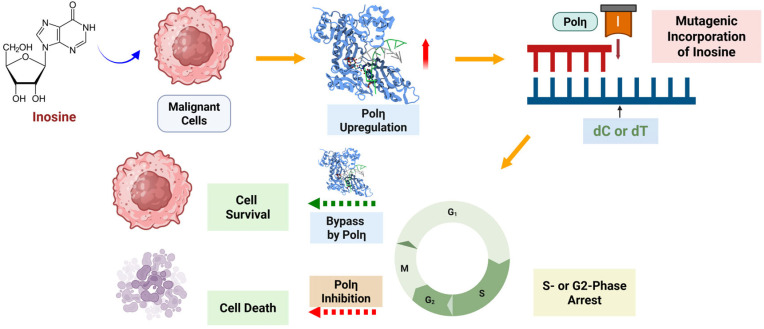
Overview of the role of polη in the mutagenic incorporation of inosine, which can lead to cell cycle arrest, and the bypass of inosine, which can resolve the replication halt. Inhibition of polη might lead to the extension of cell cycle arrest caused by the mutagenic incorporation of inosine and other DNA-targeting drugs. The figure was prepared using Biorender.

Our flow cytometry data presented here showed that 5-time treatment of inosine or IMP caused an increase in the S- and G2-phase population in HCT116 cells (Figure 3), and this effect was shown only in 5x treatment, not in 1x or 3x treatments. While there is a possibility that inosine/IMP can stimulate growth or accelerate G1/S entry and cause the population distribution to shift toward S/G2, this result suggests that polη’s bypass activity over the incorporated inosine contributes to this effect among other potential causes. The accumulation of inosine was reported to cause some detrimental effects, and one of the potential effects is cell cycle arrest, specifically at S- or G2-phase as our data presented here suggested. Currently there are several FDA-approved drugs that are known to cause cell cycle arrest, including ribociclib (CDK inhibitor, G1/S arrest), docetaxel (micro-tubule inhibitor, G2/M arrest), doxorubicin (DNA damaging agent, G1, S, G2/M arrest), and methotrexate (anti-metabolite, S-phase arrest) [49,50,51,52]. However, there has not been any report that connects TLS/polη with cell cycle arrest so far, and our data suggest that polη might play a crucial role in modulating cell cycle arrest, especially at S- and G2-phase that is caused by DNA-incorporating lesions/drugs (Figure 9). Our data suggest that the cell cycle arrest caused by DNA-incorporating drugs could be extended by inhibiting polη and TLS, and this modulation of polη can be more pronounced in an environment where DNA repair is not fully functioning, such as fast-growing cells (Figure 9).

A couple of important questions should be addressed to fully understand the role polη potentially plays in the extension of cell cycle arrest such as (1) is there any universal mechanism by which polη works for the incorporation and bypass of DNA lesions including the ones generated by DNA-incorporating drugs? (2) Is the function and modulation of polη that is related to cell cycle arrest, which was shown in inosine’s case, DNA-lesion- or cell-type-specific? For example, once inosine is incorporated into DNA, as we showed here, it was shown that polη can effectively bypass it with that strand being used as a template [22]. The involvement of polη in the incorporation and bypass of inosine and other naturally occurring DNA lesions might have a significant impact on the metabolism of nucleotide analog drugs, which is crucial for drug action and resistance, and further research, including the experiment on *POLH* knock-out cells and confirmation of polη overexpression by Western blot, will give us more detailed answers on the relationship between TLS/polη and the drug action/resistance caused by nucleotide analog drugs. Also, it is important to see if TLS and polη have similar roles on the direct incorporation and bypass of other nucleotide analog drugs such as gemcitabine or 6-thiopurines.

In conclusion, our cell biological, structural, and biochemical studies presented here provide novel insights into the mutagenic potential of inosine in DNA replication and the role of polη and TLS in the incorporation of inosine and in the cell cycle arrest caused by the cellular accumulation of inosine. In response to the repeated treatment of inosine or IMP in HCT116 cells, polη was upregulated to incorporate inosine into DNA with increased mutagenicity that can cause G:C to/from A:T mutation. The crystal structures of polη complexed with ITP and dT showed that ITP can adopt both syn- and anti-conformations in the active site of polη, and this heterogeneity of ITP conformation enabled polη to incorporate ITP across incorrect dT more efficiently than GTP. Our data showed that the increased level of inosine can lead to DNA incorporation of inosine, which can then lead to S- or G2-phase cell cycle arrest as a result. Cell cycle arrest by DNA lesions including inosine and ribonucleotide could be extended when polη’s activity is modulated.

## 4. Materials and Methods

### 4.1. Expression and Purification of the Catalytic Domain of Polη

The catalytic domain of polη (1-432) was expressed and purified based on the previously published protocols with modifications [53,54,55]. In brief, 500 mL LB media supplemented with 50 μg/mL kanamycin were inoculated with 5 mL of small overnight LB culture of polη in *E. coli* BL21(DE3) competent cells and were grown at 37 °C until reaching the OD_600_ of around 0.7. After the cells were cooled down to about 16 °C and were induced with 0.3 mM isopropyl β-D-α-thiogalactopyranoside (IPTG), the cells were further grown for 20 h at 20 °C. The induced and cultured cells were harvested and pelleted by centrifugation at 5000× *g* at 4 °C for 30 min, and the pellet was resuspended in Ni–NTA column binding buffer A (50 mM sodium phosphate, pH 7.5, 500 mM NaCl, and 10% glycerol) in supplement with 1 mg/mL lysozyme, 0.25% (*v*/*v*) NP-40, 0.25% (*v*/*v*) Triton X-100, and 0.25 mM phenylmethylsulfonyl fluoride (PMSF) for lysis via sonication (30 s × 3 rounds). The total lysate was spun down at 16,000× *g* at 4 °C for 45 min, and the supernatant was filtered through a 0.22 μm syringe filter. The filtered supernatant was loaded on Ni-NTA column (GE Healthcare, Chicago, IL, USA) and purified through it. The elution fractions were analyzed via SDS-PAGE gel and pooled for further purification through the Heparin HiTrap column (GE Healthcare, Chicago, IL, USA), and Superdex-75 size exclusion chromatography (GE Healthcare, Chicago, IL, USA). The purity of the final protein product was verified by SDS-PAGE gel. The purified polη was then concentrated to 9 mg/mL and was flash-frozen in liquid nitrogen to be stored at −80 °C for future use.

### 4.2. HCT116 Cell Culture and the Treatment with Inosine and IMP

The human colon cancer cell line, HCT116, a gift from Dr. Kyle Hadden in the Department of Pharmaceutical Sciences at the University of Connecticut, was used in this study. HCT116 cells were cultured in McCoy’s 5A medium (Gibco, 16600082, Waltham, MA, USA) supplemented with 10% heat-inactivated fetal bovine serum (Gibco, 10437028, Waltham, MA, USA), 100 units/mL penicillin, and 100 μg/mL streptomycin solution (Gibco, catalog number: 15140122, Waltham, MA, USA). The cells were grown in a humidified incubator at 37 °C with 5% CO_2_, and the growth media was replaced every 2–3 days. Cells were seeded into T-25 flasks (0.7 × 10^6^ cells per flask) in triplicate and treated with 10 μM and 100 μM inosine and IMP along with DMSO as a negative control. Following incubation for 72 h, cells were harvested for RNA extraction.

### 4.3. Gene Expression Monitoring via Quantitative Reverse Transcription PCR (RT-qPCR)

The total RNA was extracted from HCT116 cells using Qiagen’s RNeasy Micro Kit (Qiagen, 74004, Hilden, Germany) according to the manufacturer’s protocol. RNA concentrations were measured using Nanodrop Spectrophotometer (Thermo Fisher Scientific, Waltham, MA, USA). One microgram of the total RNA was reverse transcribed using iScript Reverse Transcription Supermix (Bio-Rad, 1708841, Hercules, CA, USA) to prepare cDNA. Samples were prepared with Universal SYBR Green Supermix (Bio-Rad, 1725271, Hercules, CA, USA), and RT-qPCR was performed using the ABI 7500 Fast Real-time PCR system (Applied Biosystems, Carlsbad, CA, USA). The following thermal protocol was used: 95 °C for 2 min for initial denaturation, then 40 cycles of amplification (95 °C for 15 s and 60 °C for 60 s). RNA levels of the samples were normalized to the housekeeping gene β-actin RNA expression. The fold change in expression was calculated using the 2^−Δ∆CT^ method (triplicates). One-way ANOVA followed by Dunnett’s multiple comparisons test was performed using GraphPad Software, version 10.0.0 (Dotmatics, Boston, MA, USA). *p*-values from one-way ANOVA are less than 0.0001 for all the genes tested, except for polκ, whose *p*-value is 0.002. The sequences of the primers used are listed below (Table 3).

### 4.4. Flow Cytometry Experiments on HCT116 Cells Treated with Inosine and IMP

HCT116 cells were cultured and treated as described above. Cells were harvested, washed with ice cold PBS, and centrifuged at 1000× *g* for 5 min. Cells were fixed in 60% ethanol for at least 4 h at 4 °C. Cells are then centrifuged at 1000× *g* for 5 min and washed with PBS. Cells are stained with a solution of 20 μg/mL propidium iodide (Millipore Sigma, 537059, St. Louis, MO, USA) 200 μg/mL RNase A (Millipore Sigma, 10109142001, St. Louis, MO, USA), and 0.1% (*v*/*v*) Triton X-100 (Millipore Sigma, 648466, St. Louis, MO, USA) in PBS at room temperature for 30 min. A total of 20,000 events were collected, and cell cycles were analyzed using Dean-Jett Fox Model. Cells were analyzed using BD LSRFortessa X-20 Cell Analyzer (BD Biosciences, Franklin Lakes, NJ, USA) and data were collected using BD FACS Diva (version 8.0.1) (BD Biosciences, Franklin Lakes, NJ, USA) and curated using FlowJo (version 10.10.0) (BD Biosciences, Franklin Lakes, NJ, USA).

### 4.5. Polη-dC/dT:ITP Complex Crystallization, Data Collection, and Refinement

To obtain ternary crystal structure of polη complexed with the incoming ITP paired with templating undamaged DNAs containing dC or dT, polη-dC/dT-containing DNA-ITP complex crystals were grown in a crystallization solution containing 100 mM MES pH 6.5, 14–23% (*v*/*v*) PEG2000 MME, and 5 mM calcium chloride. The undamaged dC or dT containing 12-mer DNA (5′-CAT[C/T]CTCACACT-3′) and the 8-mer primer (5′-AGTGTGAG-3′) were synthesized by Integrated DNA Technologies (Coralville, IA, USA). The template and the primer oligonucleotides were annealed in hybridization buffer (10 mM Tris-HCl pH 7.5, 1 mM EDTA) at 90 °C for 5 min followed by the slow cooling to room temperature. Polη was incubated with the annealed double stranded recessed DNA with 1:1.5 molar ratio. Subsequently, 10-fold molar excess of ITP (Sigma, St. Louis, MO, USA) was added to the binary complex of polη-DNA. Crystals grew to a full size with diffraction quality in 2–3 weeks. The crystals were then cryoprotected in the mother liquor supplemented with 20% (*v*/*v*) glycerol and were flash-frozen in liquid nitrogen for data collection. Diffraction data were collected at 100 K at the beamline 17-ID-1 (AMX) at the Advanced Photon Source, Argonne National Laboratory. All diffraction data were processed following the data collection onsite using XDS [56,57] and aimless [58,59], and the structure was solved by molecular replacement using Molrep [60] with polη structure with an undamaged DNA (PDB ID 4O3N) as a search model. The model was built using COOT [61] and refined using PHENIX [62]. All the crystallographic figures were generated using Chimera [63].

### 4.6. Steady-State Kinetics of Single Nucleotide Incorporation of ITP Opposite Templating dC/dT by Polη

Steady-state kinetic parameters for single nucleotide insertion across undamaged dC and dT by polη were measured as described previously, with slight modification [25,54]. To describe briefly, the oligonucleotides for kinetic assays, the FAM-labeled primer (5′-FAM/GGGGGAAGGATTC-3′) and the undamaged dC- or dT-containing templates (5′-TTCAT[C/T]GAATCCTTCCCCC-3′) were synthesized by Integrated DNA Technologies (Coralville, IA, USA). Templating DNA containing the undamaged dC or dT was annealed with the primer in hybridization buffer (10 mM Tris-HCl pH 7.5, 1 mM EDTA) at 90 °C for 5 min followed by slow cooling to room temperature. Enzyme activities of polη were measured using the reaction mixture containing 40 mM Tris-HCl pH 7.5, 60 mM KCl, 10 mM dithiothreitol, 250 μg/mL bovine serum albumin, 2.5% (*v*/*v*) glycerol, 5 mM MgCl_2_, 80 nM primer/template DNA, and 8 different concentrations of incoming nucleotides including ITP. The enzymatic reaction time and the concentration of polη were adjusted for each experiment for the product formation of about 20% or less to prevent end-product inhibition and substrate depletion from interfering with accurate velocity measurement. The reactions were initiated by the addition of the incoming ribonucleotide and stopped with gel-loading buffer (95% (*v*/*v*) formamide with 20 mM EDTA, 45 mM Tris-borate, 0.1% (*w*/*v*) bromophenol blue, 0.1% (*w*/*v*) xylene cyanol), and the quenched samples were separated on 18% denaturing polyacrylamide gels. The gels were analyzed using ChemiDoc MP (version 3.0.1.14) and Image Lab Software (version 6.1) (BioRad, Hercules, CA, USA) to quantify product formation. The kinetic parameters (*k*_cat_ and *K*_m_) were determined by fitting reaction rate over ITP concentrations to the Michaelis–Menten and Lineweaver–Burk equations. Each experiment was repeated three times to measure the average of the kinetic parameters along with the standard deviation. The efficiency of nucleotide insertion was calculated as *k*_cat_/*K*_m_. The relative frequency of ITP incorporation opposite dC or dT was determined as *f* = (*k*_cat_/*K*_m_)_[dT:ITP]_/(*k*_cat_/*K*_m_)_[dC:ITP]_.

## Figures and Tables

**Table 1:** Kinetic parameters for ribonucleotide incorporation opposite dC and dT by polη.

Template:(d)NTP	*K*_m_ (μM)	*k*_cat_ (10^−3^s^−1^)	*k*_cat_/*K*_m_ (10^−3^s^−1^μM^−1^)	*f* ^a^	Replication Fidelity
**polη**					
dC:ITP	272.0 ± 16.3	5.5 ± 0.5	0.02	1	18.2
dT:ITP	1683.9 ± 84.8	1.8 ± 0.2	0.0011	0.055	1
dC:dITP ^b^	25.0 ± 4.8	176.0 ± 10.9	7.17	1	13.7
dT:dITP ^b^	85.3 ± 6.3	44.5 ± 3.3	0.52	0.073	1
dC:GTP	385.3 ± 32.3	2.8 ± 0.6	0.0073	1	
dT:GTP	-	-	N/D	-	
dC:ATP	-	-	N/D	-	
dT:ATP	476.3 ± 27.9	2.3 ± 0.4	0.0048	1	
dC:dGTP ^b^	9.8 ± 1.4	180.9 ± 5.8	18.5	1	185
dT:dATP ^b^	7.9 ± 0.6	157.9 ± 4.9	19.9	1	181
HX:dCTP ^c^	4.6 ± 0.4	170.5 ± 4.1	37.4	1	69
HX:dTTP ^c^	21.9 ± 1.4	11.7 ± 0.2	0.54	0.014	1

^a^ Relative efficiency: (*k*_cat_/*K*_m_)_[(d)NTP:Test]_/(*k*_cat_/*K*_m_)_[(d)NTP:Correct]_. ^b^ Averill et al. [45] ^c^ Jung et al. [22].

**Table 2:** Data collection and refinement statistics.

PDB CODE	Polη	Polη dT:	Polη dT:
	dC:ITP	*anti*-ITP	*syn*-ITP
	(8G8H)	(8G8J)	(8GBF)
**Data Collection**			
space group	*P*6_1_	*P*6_1_	*P*6_1_
Cell Constants			
a (Å)	99.27	98.90	99.30
b	99.27	98.90	99.30
c	81.76	81.22	81.61
α (°)	90.00	90.00	90.00
β	90.00	90.00	90.00
γ	120.00	120.00	120.00
resolution (Å) ^a^	43.0–1.64	42.8–1.74	49.6–2.11
	(1.67–1.64)	(1.77–1.74)	(2.15–2.11)
R_merge_ ^b^ (%)	0.026 (0.651)	0.027 (0.662)	0.045 (0.537)
<I/σ>	14.5 (1.2)	15.6 (1.2)	10.9 (1.5)
CC_1/2_	0.505	0.455	0.489
completeness (%)	97.5 (100)	86.0 (99.9)	86.0 (93.2)
redundancy	11.9 (11.2)	12.0 (12.6)	10.5 (9.7)
**Refinement**			
R_work_^c^/R_free_^d^ (%)	20.9/24.1	21.1/24.5	20.5/25.7
unique reflections	54113	39698	25501
Mean B Factor (Å^2^)			
protein	33.75	35.84	37.96
ligand	37.35	42.23	45.91
solvent	37.00	37.82	37.50
Ramachandran Plot			
most favored (%)	97.7	97.9	97.4
add. allowed (%)	1.2	1.7	2.2
RMSD			
bond lengths (Å)	0.008	0.009	0.008
bond angles (degree)	1.155	1.176	1.057

^a^ Values in parentheses are for the highest resolution shell. ^b^ *R*_merge_ = Σ|I−<I>|/ΣI where I is the integrated intensity of a given reflection. ^c^ *R*_work_ = Σ|F(obs)−F(calc)|/ΣF(obs). ^d^ *R*_free_ = Σ|F(obs)−F(calc)|/ΣF(obs), calculated using 5% of the data.

**Table 3:** The sequences of the primers for RT-qPCR.

Gene Name	Forward	Reverse
*β-actin*	GGCACCCAGCACAATGAAG	GCCGATCCACACGGAGTACT
*POLK*	GTTCTAGTCTCCCAAGCAAGTC	GCTGGCGGTATTCTTGTCTAA
*POLH*	CATGGAAGGGTGGTGGAATAA	AGCATCATCTGCCCACATAC
*POLD*	CCAGACCCTCAAGGTACAAAC	CTGCTTGGACTGGAATGAAGA
*POLI*	GGTGGTTACCTGCAACTATGA	GGGTCAGGTCTTCTCCATTAAC
*GMPS*	ATGGCTCTGTGCAACGGAG	CCTCACTCTTCGGTCTATGACT
*SHMT-1*	TTGCCTCGGAGAATTTCGCC	GTCCCATAGTATCTCTGG
*SHMT-2*	CCCTTCTGCAACCTCACGAC	TGAGCTTATAGGGCATAGACTCG
*POLB*	AGCACTAGGGGGTGGAAAGG	CATCATTGGGCCCCCTTTTT

## Data Availability

The atomic coordinates of polη-DNA complexed with ITP have been deposited in the Protein Data Bank with the following accession codes: polη-dC:ITP (PDB Code: 8G8H), polη-dT:ITP (anti) (PDB Code: 8G8J), and polη-dT:ITP (syn) (PDB Code: 8GBF).
